# A prior‐information‐based automatic segmentation method for the clinical target volume in adaptive radiotherapy of cervical cancer

**DOI:** 10.1002/acm2.14350

**Published:** 2024-03-28

**Authors:** Xuanhe Wang, Yankui Chang, Xi Pei, Xie George Xu

**Affiliations:** ^1^ School of Nuclear Science and Technology University of Science and Technology of China Hefei China; ^2^ Anhui Wisdom Technology Company Ltmited Hefei China; ^3^ Department of Radiation Oncology The First Affiliated Hospital of University of Science and Technology of China Hefei China

**Keywords:** adaptive radiotherapy, cervical cancer radiotherapy, clinical target volume, deep learning

## Abstract

**Objective:**

Adaptive planning to accommodate anatomic changes during treatment often requires repeated segmentation. In this study, prior patient‐specific data was integrateda into a registration‐guided multi‐channel multi‐path (Rg‐MCMP) segmentation framework to improve the accuracy of repeated clinical target volume (CTV) segmentation.

**Methods:**

This study was based on CT image datasets for a total of 90 cervical cancer patients who received two courses of radiotherapy. A total of 15 patients were selected randomly as the test set. In the Rg‐MCMP segmentation framework, the first‐course CT images (CT1) were registered to second‐course CT images (CT2) to yield aligned CT images (aCT1), and the CTV in the first course (CTV1) was propagated to yield aligned CTV contours (aCTV1). Then, aCT1, aCTV1, and CT2 were combined as the inputs for 3D U‐Net consisting of a channel‐based multi‐path feature extraction network. The performance of the Rg‐MCMP segmentation framework was evaluated and compared with the single‐channel single‐path model (SCSP), the standalone registration methods, and the registration‐guided multi‐channel single‐path (Rg‐MCSP) model. The Dice similarity coefficient (DSC), 95% Hausdorff distance (HD95), and average surface distance (ASD) were used as the metrics.

**Results:**

The average DSC of CTV for the deformable image DIR‐MCMP model was found to be 0.892, greater than that of the standalone DIR (0.856), SCSP (0.837), and DIR‐MCSP (0.877), which were improvements of 4.2%, 6.6%, and 1.7%, respectively. Similarly, the rigid body DIR‐MCMP model yielded an average DSC of 0.875, which exceeded standalone RB (0.787), SCSP (0.837), and registration‐guided multi‐channel single‐path (0.848), which were improvements of 11.2%, 4.5%, and 3.2%, respectively. These improvements in DSC were statistically significant (*p* < 0.05).

**Conclusion:**

The proposed Rg‐MCMP framework achieved excellent accuracy in CTV segmentation as part of the adaptive radiotherapy workflow.

## 1 INTRODUCTION

Cervical cancer ranks fourth in both incidence and mortality rates among women.^1^ Over the past four decades, the survival rates of cervical cancer patients have not significantly improved. Furthermore, the death rate continues to increase at an approximate rate of 1% annually.^1^ These trends highlight a notable gap in therapeutic advancements. Radiotherapy, used either as a postoperative adjuvant therapy or a standalone treatment, has shown significant potential for treating cervical cancer.^2^

The accurate segmentation of the clinical target volume (CTV) for a cervical cancer patient is the foundation for ensuring the success of radiotherapy.^3^ However, the anatomical changes in tumor shape and size during treatment can cause excessive radiation doses to dangerous organs (OAR) or a decrease in the planned radiation dose to the target area.^4^ To address this issue, Yan et al. proposed a closed‐loop radiotherapy treatment planning process, now known as adaptive radiation therapy (ART), that calls for adjusting the initial treatment plan according to individual patient changes during the treatment.^5^ However, implementing ART as part of the radiotherapy workflow requires considerable resources, particularly repeated contour modifications and plan evaluations.

Recently, deep‐learning‐based (DL) methods using Convolutional neural networks (CNN) have been applied to the segmentation of images of a variety of cancers.^6^, ^7^, ^8^ In terms of cervical cancer, it has shown comparable performance to the expert delineation for some cervical cancer segmentation.^9^, ^10^, ^11^, ^12^, ^13^, ^14^ However, the performance of these auto‐segmentation networks is highly dependent on the training dataset, which is affected by the difference in physician contouring styles and the anatomic structure differences among patients.^15^, ^16^ In order to generalize the trained network to a variety of unseen patient cases, network training is usually based on a large enough training set of different patient cases. In this case, the network may achieve good overall performance across different patients in application, but may not achieve the best possible performance for a specific patient.^17^ However, for ART, a sequence of longitudinal image data for a specific patient needs to be contoured during the whole treatment process, and some prior CT and contour information is usually available from the initial treatment planning and a few early fractions. Such patient‐specific information is essential for plan adaptation. The registration method is an effective way to propagate the contours from the initial treatment plan, which utilizes the prior information of the patient, but its accuracy is limited by the registration algorithm,^18^ which might perform poorly on deformable structures. Therefore, we hold the opinion that incorporating prior patient‐specific information into the automatic segmentation model through registration methods and developing a patient‐specific network may improve the auto‐segmentation accuracy for subsequent images in ART.^19^, ^20^, ^21^, ^22^

To our knowledge, the most common approach for incorporating prior knowledge into the segmentation model involves channel stacking at the input proposed by Ma et al.^23^ However, no examples of applying a channel‐based multi‐path feature extraction network for the automatic segmentation of CTV in cervical cancer have been presented. Therefore, in this study, registration methods are combined with this network architecture to achieve more accurate segmentation on the CT images of subsequent fractions. We propose a registration‐guided multi‐channel multi‐path (Rg‐MCMP) model framework, which merges the aligned contour and image produced by the registration algorithm with the subsequent fractional image as the inputs of Rg‐MCMP. To assess the efficacy of our model, Rg‐MCMP is compared with other models, including standalone registration, the standalone single‐path single‐channel (SCSP) model, and the registration‐guided multi‐channel single‐path (Rg‐MCSP) model.

## 2 METHODS

### 2.1 Patient datasets

**FIGURE 1:**
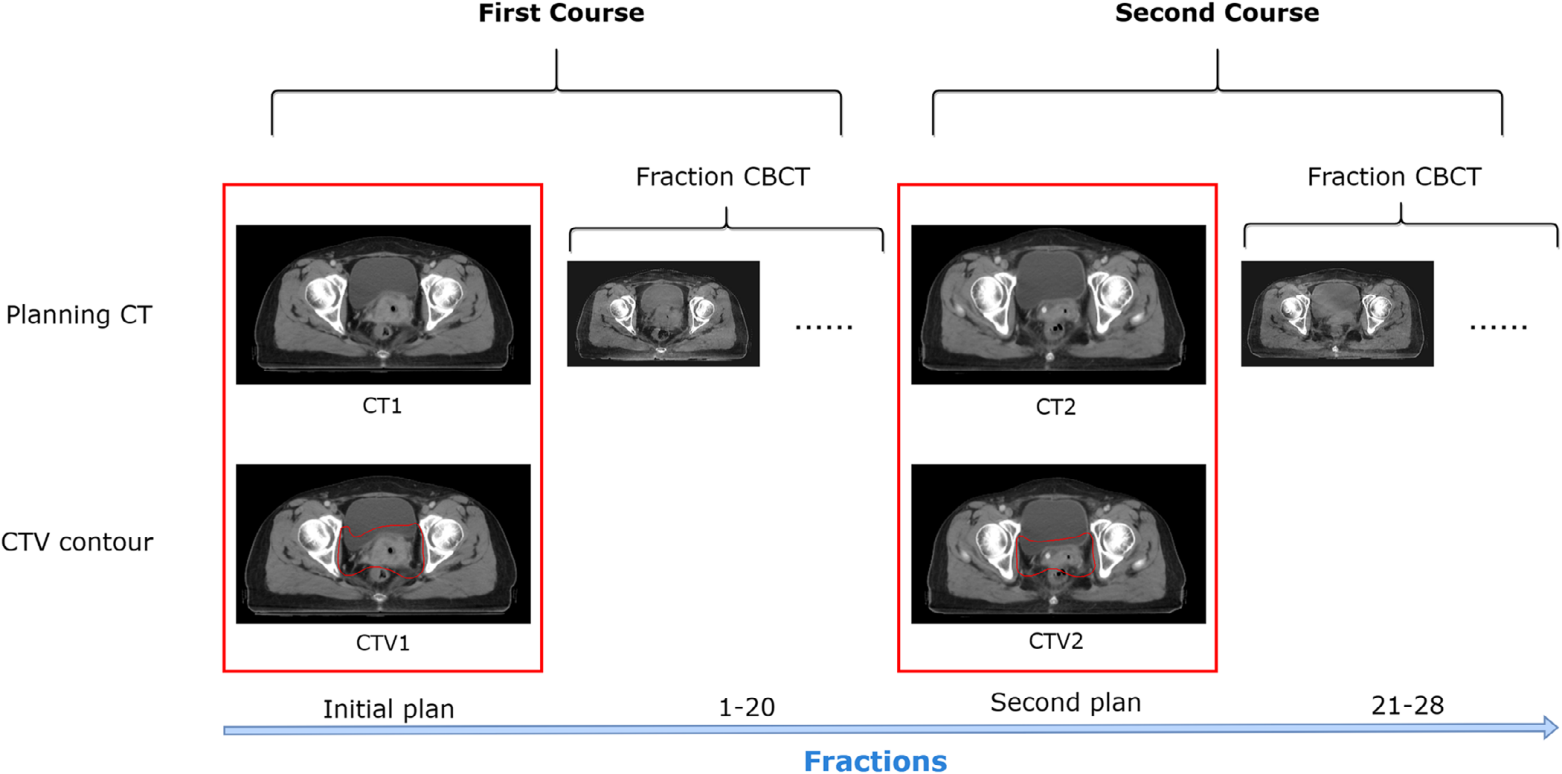
Data acquisition of paired CT and CTV contours. The data in the red box were collected in this study. CTV, clinical target volume.

This study utilized data retrospectively collected from 90 patients with cervical cancer. All patients received two‐course intensity‐modulated radiotherapy (IMRT) treatment. The first course consisted of 20 fractions, including an initial planning CT simulation before the first course treatment and the 20 fractioned CBCT simulations during the first fractional treatment, and the second course consisted of 8 fractions, containing a new CT simulation before second course treatment and the 8 fractioned CBCT simulations during the second course treatment. The total prescribed dose was 50.4 Gy, with 1.8 Gy/fraction. As shown in Figure 1, each patient had 2 sets of CT images and CTV contours: (1) CT image and the corresponding CTV contour for the first course and (2) CT image and corresponding CTV contour for the second course. Among these 90 patients, 60 patients were randomly selected for the training set, 15 patients for the validation set, and the remaining 15 patients for the test set. The CTV contours were delineated manually by senior radiation oncologists with more than 10 years of clinical experience. Their delineations conformed to the recommendations for cervical cancer radiotherapy target area contours by the Radiation Therapy Oncology Group (RTOG) 0418 guidelines,^24^ started from the bifurcation of the common iliac artery and included the primary tumor, uterus, appendix, part of the vagina (the upper half or two‐thirds of the vagina according to the primary tumor), and the pelvic lymph nodes (common iliac, external iliac, internal iliac, obturator, and presacral). The CT images were obtained on a PHILIPS BrillianceTM Bigbore CT, which had a bore with a diameter of 85 cm. The plane resolution of the CT ranged from 0.962 mm × 0.962 mm to 1.365 mm ×1.365 mm, and the slice thickness was 5 mm. The CBCT images were obtained from a Halcyon 2.0 system (Varian, USA), with plane resolution ranging from 0.908 mm ×0.908 mm to 1.035 mm ×1.035 mm and slice thickness of 2 mm.

### 2.2 Image preprocessing

For each patient in this dataset, a series of preprocessing steps were implemented. First, the first course planning CT (CT1) was registered to the second course planning CT (CT2) by either rigid body (RB) registration or deformable image registration (DIR), and the planning contours were subsequently propagated. Second, the 48 axial CT slices were extracted to ensure that all CTVs were included within this range. To focus on organs and suppress the background information, we cropped the regions of interest according to the maximum body contours of registered CT1 and CT2. Finally, due to computer memory limitations, the CT images were resampled using linear interpolation, and the CTV labels were resampled using nearest neighbor interpolation. The final size of the resampled CT images and CTV labels was 192 × 160 × 48 pixels. The values of the CT image were truncated with the threshold (−1000, 1500) and linearly normalized to (0,1).

### 2.3 Registration‐guided multi‐channel multi‐path (Rg‐MCMP) segmentation framework

**FIGURE 2:**
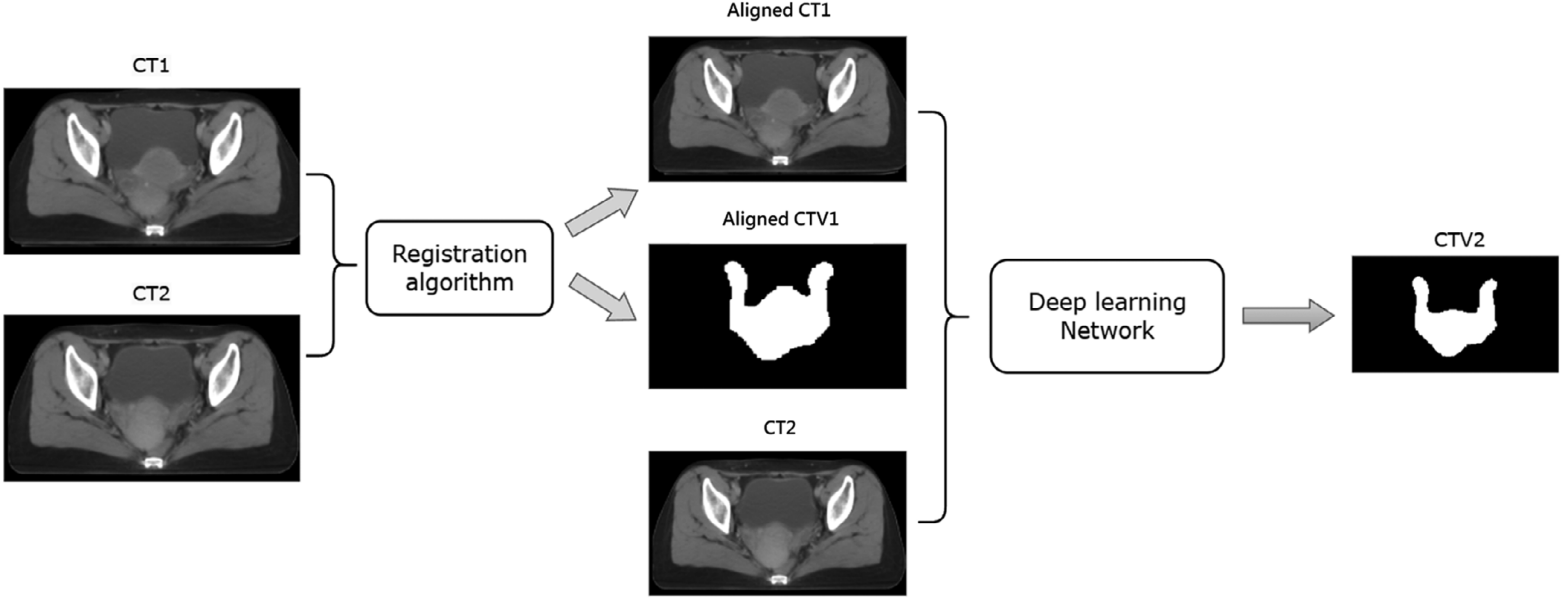
Registration‐guided multi‐channel multi‐path segmentation framework. Aligned CT1, aligned first course plan CT; Aligned CTV1, aligned clinical target volume; CT1, first course plan CT; CT2, second course plan CT; CTV2, clinical target volume of the second course of treatment.

In this study, we formed a registration‐based multi‐channel multi‐path (Rg‐MCMP) framework for CTV automatic segmentation of cervical cancer, as shown in Figure 2. This framework comprised two phases: (1) contour propagation via registration and (2) the implementation of a multi‐channel multi‐path segmentation model. In the first phase, the first course CT images (CT1) were registered to the CT2 images to yield aligned CT images (aCT1), and the CTV1 contours were propagated to yield the aligned CTV contours (aCTV1). In the second phase, aCT1, aCTV1, and CT2 were merged as three input channels to improve CTV segmentation accuracy.

#### 2.3.1 Image registration algorithms

In this study, we employed two image registration algorithms: RB and DIR. These algorithms establish voxel‐to‐voxel correspondences between a fixed image I_f_ and a moving image I_m_.

Utilizing the ITK 4.13 library,^25^ the RB algorithm transforms I_m_ into I_f_ through RB transformation. The stochastic gradient descent was chosen as the optimizer with an initial learning rate of 8 and Mattes mutual information as the metric. The algorithm terminated either after 500 optimization iterations or upon reaching a minimum step size of 0.001. The DIR method was formed by the Elastix B‐spline registration method,^26^ which had undergone multiple iterations to generate the final voxelized deformation vector field (DVF). A pyramid multiscale strategy was incorporated that consisted of a four‐level hierarchy and an optimization grid resolution of 16.

#### 2.3.2 Network architecture

**FIGURE 3:**
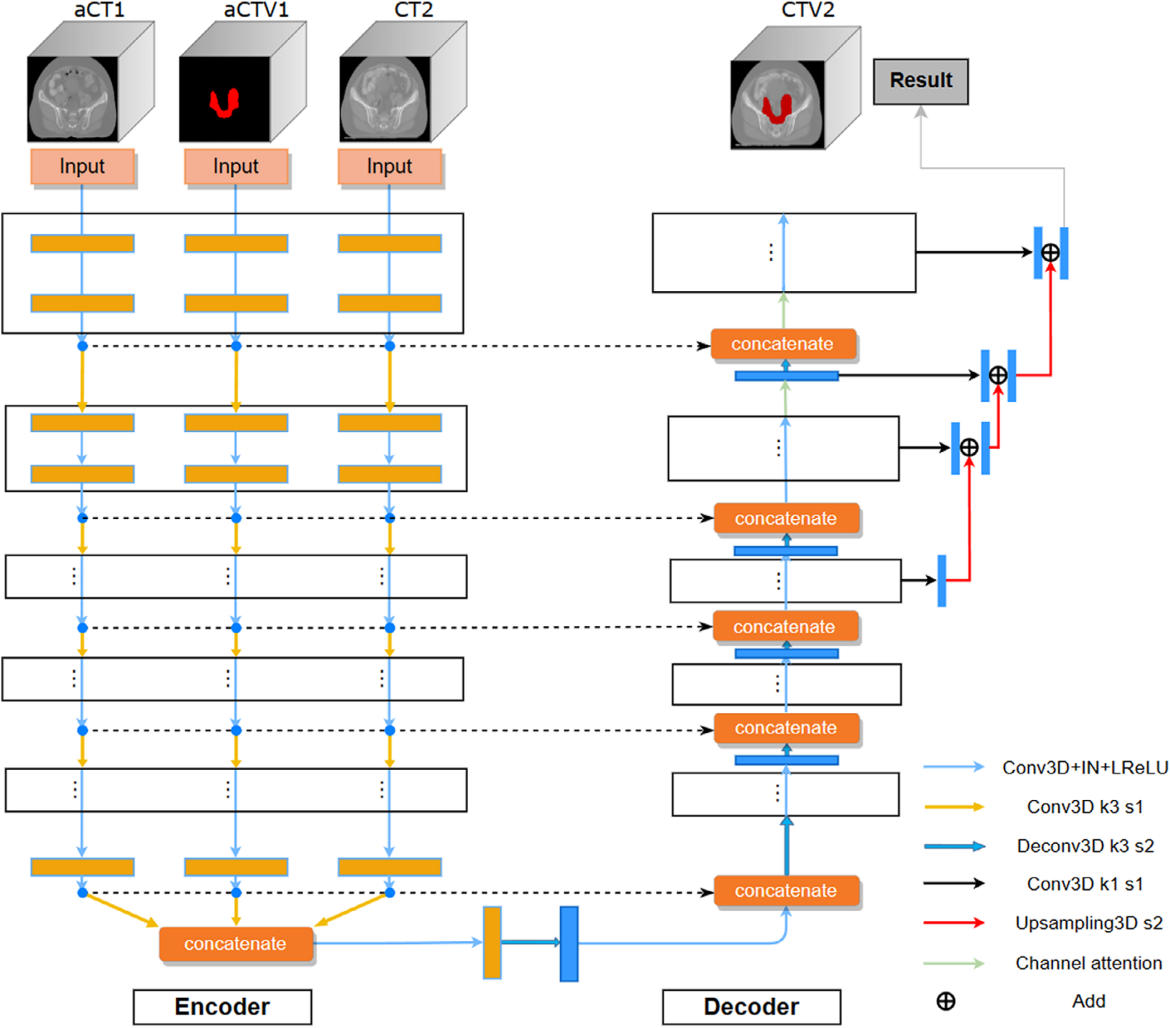
Network framework. The registered contour and two sets of CT images were used together as the input of the network. aCT1, aligned first course plan CT; aCTV1, aligned target contour; CT2, second course plan CT; CTV2, target contour of the second course of treatment.

Figure 3 illustrates the architecture of the Rg‐MCMP model. The input layer consists of three channels corresponding to the aligned CT1 (registered first course plan CT), aligned CTV1 (registered target contour), and CT2. This model was developed on the 3D U‐Net^27^ and was composed of an encoder and a decoder. The encoder contained three independent paths, each responsible for its unique feature extraction. These independent paths were intended to extract image features from their respective inputs without early‐stage feature merging, thus preventing the loss of each image's unique features. Although each set of input data had an independent encoding path, the entire network was trained simultaneously. The decoder used long skip connections to connect the output of each convolution module with the feature mapping from the encoder of the same depth. Copying the additional feature mappings from each encoding path made it easier for the decoding path to recover image information lost during downsampling.^28^

Because the cross‐sectional dimensions of the three‐dimensional input CT image were larger than the axial dimension, only the lateral dimensions underwent downsampling in the encoder's first and last steps, while the axial size was unchanged. Each depth level of the encoder consisted of two three‐dimensional convolutional modules. Each convolutional module comprised a convolution layer with a kernel of 3 × 3 × 3, an instance normalization layer, and an LReLU (*α* = 0.2) activation layer. The downsampling layer was completed by using a convolution layer with a step size of 2, which reduced the size of the image by half and doubled the number of channels to compensate for the information loss caused by downsampling. In the decoder, each level contained an upsampling layer and a pair of convolution modules, where upsampling was achieved through 3D deconvolution. The final three segmentation blocks employed a 1 × 1 × 1 convolution layer to convert the feature tensor into a probability tensor. All results were then combined through a linear interpolation upsampling operation to enhance the precision of the segmentation results in the process called deep supervision. Moreover, the model also utilized the channel attention (CA) module,^29^ which assigned varying weights to the input feature map channels, highlighting critical channels while diminishing the less significant ones.

### 2.4 Experimental design

We implemented two versions of the Rg‐MCMP segmentation framework: RB‐MCMP, which employed RB for contour propagation and used the aligned CT images and aligned contours as guidance, and DIR‐MCMP, which employed B‐spline DIR and used the contours and CT images generated through DIR as guidance. Both implementations were trained using the same ground truth (GT) but different registration guidance inputs. The performances of RB‐MCMP and DIR‐MCMP were evaluated against standalone registration (either RB/DIR), the single‐channel single‐path model (SCSP) (without registration guidance), and the Rg‐MCSP model, which included the RB registration‐guided multi‐channel single‐path model (RB‐MCSP) model and DIR‐guided multi‐channel single‐path model (DIR‐MCSP). The implementation details of the comparison experiment are shown in Table 1. Moreover, the RB or DIR simulated the clinical registration process. The SCSP mirrored the clinician's process of re‐segmentation of the target area of fractional images and could also be used to study the effectiveness of multi‐channel multi‐path extraction strategies. By comparing the proposed framework with the Rg‐MCSP, we can understand the influence of the multi‐path independent feature extraction network on the encoder path. The SCSP model and Rg‐MCSP used the same optimization methods and training strategies as the Rg‐MCMP model, with only slight differences in architecture, as detailed below. The SCSP model used CT2 as its sole input, while the Rg‐MCSP model combined aligned CT1 and aligned CTV1, with CT2 at the inputs, feeding them into the network as multiple channels. Both models had only a single path for extracting features in the encoder path, but their decoders were the same as that of the Rg‐MCMP. Since our model was a multi‐channel input with a multi‐path independent feature extraction strategy modified on the basis of the SCSP model, the validity of our chosen basic model (SCSP) must be validated. Therefore, we also compared the performance of SCSP to that of 3D U‐Net.^27^ Both SCSP and 3D U‐Net adopted the same training strategies. Comparative results are presented in Section 3. The primary metric employed was the Dice similarity coefficient (DSC), which quantifies the spatial overlap between network predictions and manual contours. Additionally, the 95th percentile Hausdorff distance (HD95) and average surface distance (ASD) were utilized as evaluation metrics.

**TABLE 1:** The implementation details of the comparison experiment.

	Method	Input	Architecture
Registration	RB	CT1	CT1 were registered to the CT2 by Rigid Body registration
	DIR	CT1	CT1 were registered to the CT2 by B‐spline Deformable Image registration
Direction DL	3D U‐Net	CT2	Standard 3D U‐Net architecture; Used CT2 as its sole input without registration guidance
	SCSP	CT2	Variant of 3D U‐Net; Used CT2 as its sole input without registration guidance
Registration‐guided multi‐channel single‐path model	RB‐MCSP	(RB guided) aCT1, aCTV1, and CT2 as multi‐channel input	A variant based on the SCSP architecture with only a single path for extracting features; Guided by rigid body registration.
	DIR‐MCSP	(DIR guided) aCT1, aCTV1, and CT2 as multi‐channel input	A variant based on the SCSP architecture with only a single path for extracting features; Guided by deformable image registration.
Registration‐guided multi‐channel multi‐path model	RB‐MCMP	(RB guided) aCT1, aCTV1, and CT2 as multi‐channel input	A variant based on the SCSP architecture, incorporating a multi‐path independent feature extraction strategy; Guided by rigid body registration.
	DIR‐MCMP	(DIR guided) aCT1, aCTV1, and CT2 as multi‐channel input	A variant based on the SCSP architecture, incorporating a multi‐path independent feature extraction strategy; Guided by deformable image registration.

Abbreviations: aCT1, aligned first course plan CT; aCTV1, aligned target contour; CT1, first course plan CT; CT2, second course plan CT; CTV2, target contour of the second course of treatment; DIR, deformable image registration; DIR‐MCMP, DIR‐guided multi‐channel multi‐path model; DIR‐MCSP, DIR‐guided multi‐channel single‐path model; DL, deep learning; RB, rigid body; RB‐MCMP, RB‐guided multi‐channel multi‐path model; RB‐MCSP, RB‐guided multi‐channel single‐path model; SCSP, single‐channel single‐path model.

Experiments were conducted on a Windows 10 (x64) workstation equipped with an NVIDIA GeForce 2080 Ti GPU with 12 GB of memory. All models for this study were implemented using Python (version 3.8) and the Keras library (version 2.4.3). In semantic segmentation tasks, commonly used loss functions include the Dice loss function and the cross‐entropy loss function. This study combined these two types of loss functions to serve as the optimization objectives for the organ segmentation neural network. The cross‐entropy loss function used in this article is presented in Equation (1), and the Dice loss function is presented in Equation (2).

(1) $$L_{ce}=\frac{1}{N}\sum_{i=1}^{N}\sum_{k=1}^{K}\left(-y_{i,k}\times \log\left(p_{i,k}\right)\right)$$

(2) $$\begin{array}{ccc} L_{dice} & = & 1-\frac{1}{K}\sum_{k=1}^{\kappa }L_{dice,k}\\ & = & 1-\frac{1}{K}\sum_{k=1}^{K}\frac{2\times \sum_{i=1}^{N}\left(p_{i,k}\times y_{i,k}\right)+\varepsilon }{\sum_{i=1}^{N}p_{i,k}+\sum_{i=1}^{N}y_{i,k}+\varepsilon } \end{array}$$

where 𝑝_𝑖,𝑘_ is the predicted probability that the voxel of sample i belongs to class k, 𝑦_𝑖,𝑘_ is the GT label (0 or 1), N is the total number of voxels in the sample, K is the number of classes and 𝜀 is a small value to prevent the denominator from being 0; 𝜀 is set to 1 in this paper.

The total loss function is shown in Equation (3), and all models use the same loss function:

(3) $$L=0.5\times L_{ce}+0.5\times L_{dice}$$

During training, the weights were initialized using the “he_normal” method.^30^ The initial learning rate was set to 0.0002. The Adam gradient descent algorithm was used to optimize the neural network parameters. The batch size was 1. The loss in the validation set was calculated once for each epoch. When the validation set loss did not decrease for 30 consecutive epochs, the learning rate was reduced to 0.2 times the original. To prevent overfitting, when the validation set loss did not decrease for 50 consecutive epochs, the training was terminated, and the model parameters with the smallest validation set loss were saved; that is, the best model on the validation set was used for the final evaluation on the test set. In particular, the same hyperparameters are applied to all deep learning models evaluated in our study, ensuring that the models' comparative performance is due to architectural differences rather than changes in hyperparameters.

## 3 RESULTS

**FIGURE 4:**
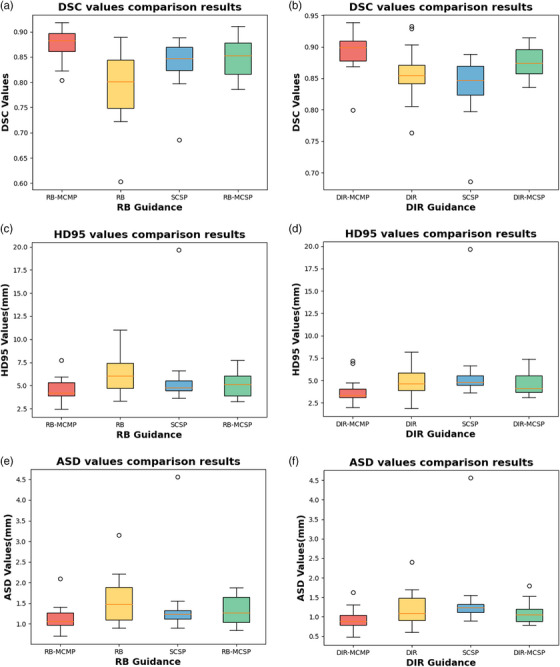
Boxplots obtained for DSC, HD95, and ASD analyses. (a) DSC values comparison results under RB registration guidance; (b) DSC values comparison results under DIR registration; (c) HD95 values comparison results under RB registration; (d) HD95 values comparison results under DIR registration; (e) ASD values comparison results under RB registration; (f) ASD values comparison results under DIR registration. ASD, average surface distance; DIR, deformable image registration, DSC, Dice similarity coefficient; HD95, 95th percentile Hausdorff distance; RB, rigid body.

The comparison results of the segmentation accuracy metrics (DSC, HD95, and ASD) are presented in Table 2 and Figure 4. The average DSC for the SCSP was found to be 0.837, which is greater than the 0.828 for 3D U‐Net, indicating the efficacy of the auto‐segmentation model used in this study.

**TABLE 2:** Comparison of segmentation accuracy between proposed methods (RB‐MCMP and DIR‐MCMP), benchmark methods (SCSP, RB, and DIR), and multi‐channel single‐path models (RB‐MCSP and DIR‐MCMP).

	Methods	DSC	HD95 (mm)	ASD (mm)
No‐guided	3D U‐Net^27^	0.828	6.04	1.54
	SCSP	0.837	5.88	1.43
RB‐guided	RB	0.787	6.23	1.56
	RB‐MCSP	0.848	5.10	1.32
	**RB‐MCMP**	**0.875**	**4.48**	**1.14**
DIR‐guided	DIR	0.856	4.86	1.19
	DIR‐MCSP	0.877	4.59	1.11
	**DIR‐MCMP**	**0.892**	**3.83**	**0.93**

*Note*: One metric is reported in one block. Higher DSC and smaller HD95 and ASD values indicate better segmentation accuracy. The 3D U‐Net was designed to prove the validity of the SCSP model.

Abbreviations: 3D U‐Net, 3D U‐Net model; ASD, average symmetric surface distance; DIR, deformable image registration; DIR‐MCMP, DIR‐guided multi‐channel multi‐path model; DIR‐MCSP, DIR‐guided multi‐channel single‐path model; DL, deep learning; DSC, Dice similarity coefficient; HD95, 95th percentile Hausdorff distance; RB, rigid body registration; RB‐MCMP, RB‐guided multi‐channel multi‐path model; RB‐MCSP, RB‐guided multi‐channel single‐path model; SCSP, single‐channel single‐path model.

For our proposed model, under the guidance of RB registration, the average DSC of CTV for RB‐MCMP was found to be 0.875, greater than that of the standalone RB (0.787) and SCSP (0.837). In addition, the mean HD95 values for RB‐MCMP, RB, and SCSP were 4.48, 6.23, and 5.88 mm, respectively, while the mean ASD values were 1.14, 1.56, and 1.43 mm, respectively. Moreover, under the guidance of DIR registration, DIR‐MCMP achieved a mean DSC of 0.892, which is greater than the 0.856 for DIR and 0.837 for SCSP. The mean HD95 values for DIR‐MCMP, SCSP, and DIR were 3.83, 5.88 and 4.86 mm, respectively. The mean ASD values were 0.93, 1.43, and 1.19 mm, respectively.

Table 2 and Figure 4 also depicts a comparative analysis of the average DSC, HD95, and ASD metrics for Rg‐MCMP and Rg‐MCSP. Generally, the Rg‐MCMP outperformed the Rg‐MCSP under both registration guidance conditions. Under the guidance of DIR registration, DIR‐MCMP achieved DSC, HD95, and ASD values of 0.892, 3.83, and 0.93 mm, respectively, compared to 0.877, 4.59 and 1.11 mm for DIR‐MCSP. Under the guidance of RB registration, the RB‐MCMP achieved DSC, HD95, and ASD values of 0.875, 4.48, and 1.14 mm, respectively, compared to 0.848, 5.10, and 1.32 mm for RB‐MCSP. This demonstrated the efficacy of the multi‐path independent feature extraction network in enhancing segmentation accuracy.

The statistical significance of the comparisons is shown in Table 3, where the *p*‐values were computed using paired *t*‐tests. The *p*‐values of DSC for the Rg‐MCMP model under the same registration guidance compared to other methods were calculated separately. Obviously, the difference between the DSC performances of the Rg‐MCMP model and the other models was statistically significant (*p* < 0.05), indicating that the Rg‐MCMP models were superior to the other models.

**TABLE 3:** The statistical significance (*p*‐value) of the superiority of Rg‐MCMP (RB‐MCMP and DIR‐MCMP) to the other methods.

Methods	Results	*p*‐Value
RB‐guided	RB‐MCMP > RB	<0.001
	RB‐MCMP > DL	<0.05
	RB‐MCMP > RB‐MCSP	<0.05
DIR‐guided	DIR‐MCMP > DIR	<0.05
	DIR‐MCMP > DL	<0.001
	DIR‐MCMP > DIR‐MCSP	<0.05
Correlation	ΔRegistration ∼ ΔRg‐MCMP: R	0.419

*Note*: Rows 1−6: *p*‐values of the DSC of Rg‐MCMP versus the DSC of other methods. Row 7: correlation coefficient R between registration accuracy and Rg‐MCMP accuracy.

Abbreviations: DIR, deformable image registration; DIR‐MCMP, DIR‐guided multi‐channel multi‐path model; DIR‐MCSP, DIR‐guided multi‐channel single‐path model; DL, deep learning; GT, ground truth; RB, rigid body; RB‐MCMP, RB‐guided multi‐channel multi‐path model; RB‐MCSP, RB‐guided multi‐channel single‐path model; ΔRegistration, the DSC of DIR minus the DSC of RB; ΔRg‐MCMP, the DSC of DIR‐MCMP minus the DSC of RB‐MCMP.

DIR outperforms RB by 8.6% in DSC, 1.37 mm in HD95, and 0.37 mm in ASD, and DIR‐MCMP outperforms RB‐MCMP by 2% in DSC, 0.65 mm in HD95, and 0.22 mm in ASD. This suggested that registration precision influenced Rg‐MCMP efficacy. To quantify the correlation between registration and Rg‐MCMP precision, the correlation coefficient between ΔRegistration (DSC difference between the two registration methods) and ΔRg‐MCMP (DSC difference between the two Rg‐MCMPs) was calculated; these are presented in Table 3. A positive correlation coefficient of *R* = 0.419 was obtained, which indicated a correlation between the accuracy of the Rg‐MCMP and the precision of the guiding registration. In other words, the higher the accuracy of registration guidance, the higher the segmentation accuracy of Rg‐MCMP model.

**FIGURE 5:**
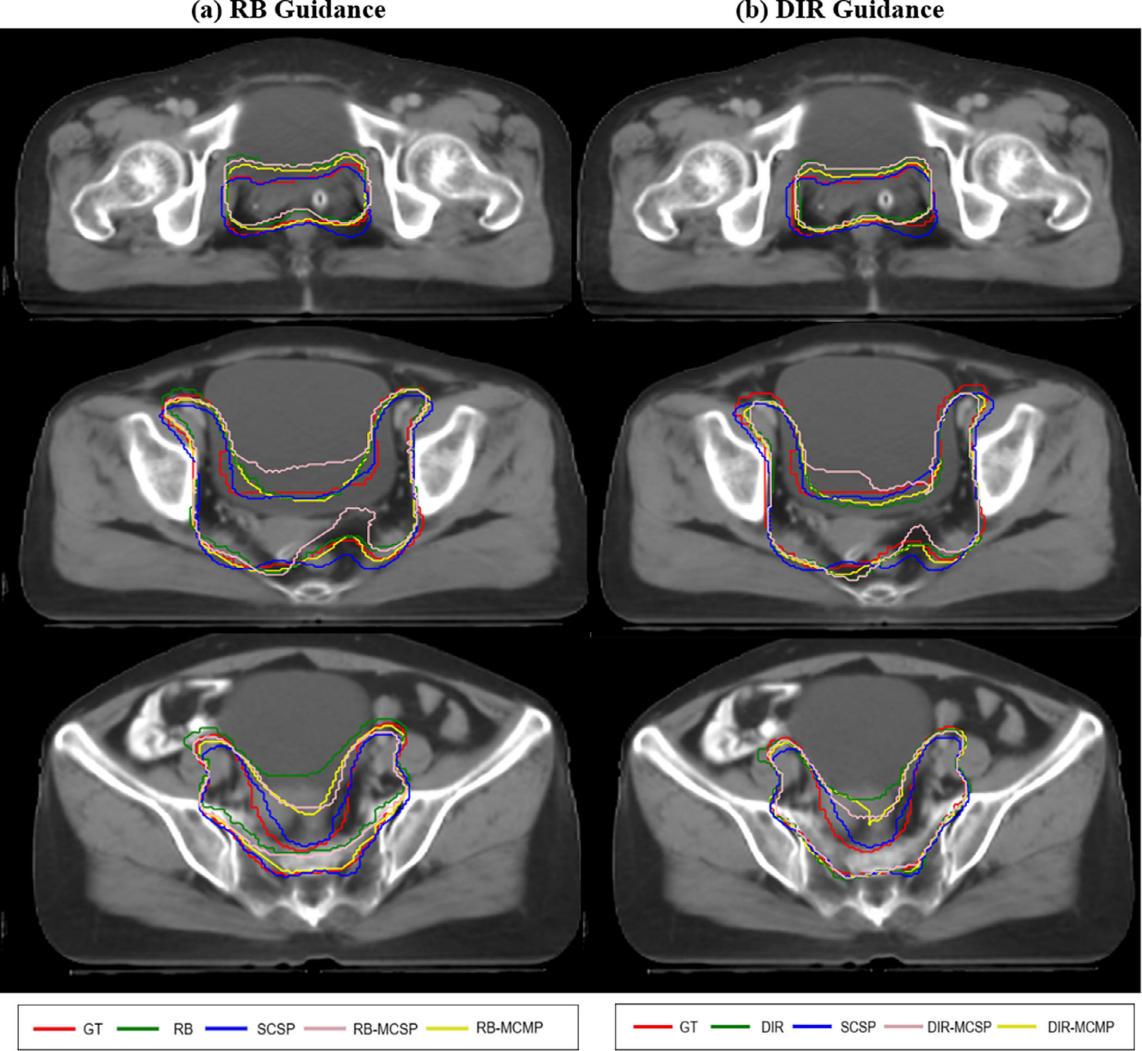
Segmentation performance analysis of target visualization (a) CT images and contours under RB registration guidance (b) CT images and contours under DIR registration guidance. DIR, deformable image registration; DIR‐MCMP, DIR‐guided multi‐channel multi‐path model; DIR‐MCSP, DIR‐guided multi‐channel single‐path model; DL, deep learning; GT, ground truth; RB, rigid body; RB‐MCMP, RB‐guided multi‐channel multi‐path model; RB‐MCSP, RB‐guided multi‐channel single‐path model.

Figure 5 displays the segmentation results of four methods employing various registration strategies for a random patient in the test set. The contours produced by the Rg‐MCMP method had the best consistency of the manually delineated contours, which demonstrated the effectiveness of our proposed method for CTV shape and position.

## 4 DISCUSSION

During adaptive treatment, the filling status and anatomical structure changes of nearby organs significantly affect the size and shape of the CTV, so repeated segmentation is required to adjust the initial treatment plan. This challenges the automatic segmentation model based on deep learning in ART.^31^ In this study, by integrating patient prior information into the segmentation model, we developed a registration‐guided multi‐path multi‐channel segmentation framework that combined the registration and DL segmentation algorithms to address CTV segmentation complexities in ART. We compared this framework with the standalone registration (RB and DIR), direct DL segmentation (SCSP), and Rg‐MCSP model. The results demonstrated the efficacy of the proposed Rg‐MCMP model in improving the auto‐segmentation accuracy, as the proposed model significantly outperformed the direct registration, direct DL segmentation, and Rg‐MCSP models. Therefore, the Rg‐MCMP framework could be an effective tool for providing more accurate and efficient automatic CTV segmentation of CT images, facilitating future ART.

Traditionally, CT image segmentation in ART has primarily been approached through contour propagation based on registration. To achieve synergy between image registration and DL segmentation in ART, Ma^23^ proposed a three‐channel adaptive automatic segmentation (TCAS) network, and the results showed that this method significantly outperforms registration. Similarly, Ma^32^ proposed an online segmentation framework based on cone beam CT (CBCT), overcoming the obstacles associated with online CBCT segmentation. Both approaches embed prior knowledge by stacking it as multiple channels at the input. Our research used the Rg‐MCSP model as a kind of reproduction of their proposed method, which was also evaluated on our dataset. According to the results in Table 2, under the guidance of RB, the DSC of RB‐MCMP was 3.2% higher than that of RB‐MCSP. Under the guidance of DIR, the DSC of DIR‐MCMP was 1.7% higher than that of DIR‐MCSP. The results indicated that under the guidance of the two registration methods, our model achieved a better prediction contour than Rg‐MCSP.

Our Rg‐MCMP combined registration with a channel‐based multi‐path feature extraction network, which could be regarded as refining registration‐propagated contours via this DL method. When integrating DL with RB registration, the RB‐MCMP improved the DSC over the standalone RB approach by 11.2%, and likewise, when integrating DL with DIR, DIR‐MCMP improved the DSC over the standalone DIR method by 4.2%. Because of the low soft‐tissue contrast of the CT images, the direct DL model could not precisely detect the boundaries of target structures without guidance, which led to its poor segmentation performance. The direct DL model (SCSP) achieved a mean DSC of only 0.837, which was improved by 4.5% (RB‐MCMP) and 6.6% (DIR‐MCMP) when combined with the guidance of registration. In addition, Rg‐MCMP significantly outperformed the other models on DSC metric in 15 test sets (*p* < 0.05). In our study, the RB‐MCMP and DIR‐MCMP versions of the proposed Rg‐MCMP model exhibited measurable enhancements in both HD95 and ASD metrics when compared with the standalone RB, standalone DIR, SCSP, and their respective MCSP versions. Specifically, the RB‐MCMP model achieved a mean HD95 value of 4.48 mm, which represents a reduction of 1.75 mm, 1.40 mm, and 0.62 mm from the standalone RB (6.23 mm), SCSP (5.88 mm), and RB‐MCSP (5.10 mm) respectively. Similarly, its ASD value of 1.14 mm is an improvement of 0.42, 0.29, and 0.18 mm over the same respective models. Furthermore, the DIR‐MCMP model demonstrated a mean HD95 value of 3.83 mm, improving upon the standalone DIR (4.86 mm) by 1.03 mm, the SCSP (5.88 mm) by 2.05 mm, and the DIR‐MCSP (4.59 mm) by 0.76 mm. The ASD value for DIR‐MCMP was 0.93 mm, which is better than the standalone DIR (1.19 mm) by 0.26 mm, SCSP (1.43 mm) by 0.50 mm, and DIR‐MCSP (1.11 mm) by 0.18 mm. These results indicate that our Rg‐MCMP model yields an improvement in precision and accuracy for HD95 and ASD metrics compared to other models. However, it is important to note that while these improvements in both metrics are present, there is no significant difference. A positive correlation was obtained between the accuracy of the model and the efficacy of the registration method. The results supported the effectiveness and generalization of the proposed Rg‐MCMP model on improving the auto‐segmentation accuracy in ART.

This study had several limitations. First, we evaluated the segmentation accuracy of Rg‐MCMP by using test cases in our in‐house dataset, which were from the same source as the training and validation data. As the quality of CT images and the contouring protocol may vary across different institutions, validating the model requires larger, diverse datasets, preferably from multiple institutions, to negate potential single‐institution biases. Second, the efficacy of our approach may vary greatly across different tumor types and locations. In this study, our focus was exclusively on cervical cancer cases, limiting our ability to assess the model's performance on other tumor target areas such as those in the head, neck, and abdomen. The multi‐channel multi‐path automatic segmentation of head and neck images will be completed in our next work.

Although our current study focused on the CTV of cervical cancer, the Rg‐MCMP framework holds potential applicability for other anatomical target structures. Considering that the target areas (such as OAR and CTV) often utilize patient‐specific clinical information to guide the extent of contouring in addition to imaging characteristics, we believe that the Rg‐MCMP approach, which incorporates patient‐specific prior information from registration and population‐derived segmentation knowledge from historical patient datasets, will be superior to just registration alone or DL alone. This assumption will be interesting to evaluate in future experiments.

Moreover, the Rg‐MCMP framework can accommodate diverse DL segmentation models and other registration algorithms. In this study, we used only a single network to demonstrate that registration guidance can improve the accuracy of CTV segmentation for ART. Many other DL segmentation models with different architectures, such as AnatomyNet^33^ and nnUnet,^34^ could also be compatible with this network framework. They may perform better than the network we used in this study. In future work, we will evaluate their performances. This framework can also be adapted to other modes of RT images, such as CBCT images or MRI images. Subsequent studies will focus on broadening the applicability of Rg‐MCMP to different anatomical sites and imaging modes to assess its utility.

## 5 CONCLUSION

In this study, we developed an Rg‐MCMP segmentation framework with an effectively improved segmentation accuracy. Experimental results showed that our proposed framework outperformed both registration methods and DL models. Furthermore, we validated the efficacy of the multi‐path feature extraction model. The results suggested that the Rg‐MCMP framework was promising for application in clinical adaptive radiotherapy.

## AUTHOR CONTRIBUTIONS

Paper idea: Xuanhe Wang, Yankui Chang, Xi Pei, and Xie George Xu. The acquisition of datasets: Yankui Chang, and Xi Pei. Autosegmentation model: Xuanhe Wang and Yankui Chang. Writing of the paper: Xuanhe Wang, Yankui Chang, and Xie George Xu.

## CONFLICT OF INTEREST STATEMENT

The authors declare no conflicts of interest.
